# Sinapic acid or its derivatives interfere with abscisic acid homeostasis during *Arabidopsis thaliana* seed germination

**DOI:** 10.1186/s12870-017-1048-9

**Published:** 2017-06-06

**Authors:** Baodi Bi, Jingliang Tang, Shuang Han, Jinggong Guo, Yuchen Miao

**Affiliations:** Institute of Plant Stress Biology, State Key Laboratory of Cotton Biology, Department of Biology, Henan University, 85 Minglun Street, Kaifeng, 475001 China

**Keywords:** Sinapic acid, Sinapic acid esters, Abscisic acid homeostasis, Seed germination, *Arabidopsis thaliana*

## Abstract

**Background:**

Sinapic acid and its esters have broad functions in different stages of seed germination and plant development and are thought to play a role in protecting against ultraviolet irradiation. To better understand the interactions between sinapic acid esters and seed germination processes in response to various stresses, we analyzed the role of the plant hormone abscisic acid (ABA) in the regulation of sinapic acid esters involved in seed germination and early seedling growth.

**Results:**

We found that exogenous sinapic acid promotes seed germination in a dose-dependent manner in *Arabidopsis thaliana*. High-performance liquid chromatography mass spectrometry analysis showed that exogenous sinapic acid increased the sinapoylcholine content of imbibed seeds. Furthermore, sinapic acid affected ABA catabolism, resulting in reduced ABA levels and increased levels of the ABA-glucose ester. Using mutants deficient in the synthesis of sinapate esters, we showed that the germination of mutant *sinapoylglucose accumulator 2* (*sng2*) and *bright trichomes 1* (*brt1*) seeds was more sensitive to ABA than the wild-type. Moreover, *Arabidopsis* mutants deficient in either *abscisic acid deficient 2* (*ABA2*) or *abscisic acid insensitive 3* (*ABI3*) displayed increased expression of the *sinapoylglucose:choline sinapoyltransferase* (*SCT*) and *sinapoylcholine esterase* (*SCE*) genes with sinapic acid treatment. This treatment also affected the accumulation of sinapoylcholine and free choline during seed germination.

**Conclusions:**

We demonstrated that sinapoylcholine, which constitutes the major phenolic component in seeds among various minor sinapate esters, affected ABA homeostasis during seed germination and early seedling growth in *Arabidopsis*. Our findings provide insights into the role of sinapic acid and its esters in regulating ABA-mediated inhibition of *Arabidopsis* seed germination in response to drought stress.

**Electronic supplementary material:**

The online version of this article (doi:10.1186/s12870-017-1048-9) contains supplementary material, which is available to authorized users.

## Background

Phenylpropanoid metabolism leads to a diverse group of compounds that are derived from the carbon skeleton of phenylalanine and are involved in plant defense, structural support, and survival [1]. Sinapic acid is a small, naturally occurring member of the phenylpropanoid family that serves as a common precursor for soluble secondary metabolites [2]. In brassicaceous plants, including *Arabidopsis thaliana*, sinapic acid is converted into a broad spectrum of *O*-ester conjugates. These abundant soluble sinapic acid esters reflect a well-known metabolic network and are produced at different stages of plant development. The accumulation of these sinapic acid esters and soluble phenylpropanoids also provides protection against ultraviolet (UV)-B stress and functions in the defense response to pathogens such as *Verticillium longisporum* in *Arabidopsis* [3, 4].

**Fig. 1:**
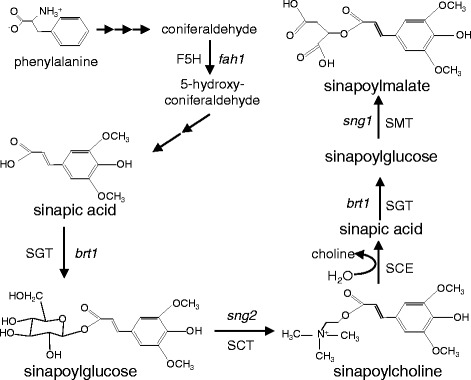
Schema of sinapate ester metabolism in *Arabidopsis*. F5H, ferulate 5-hydroxylase; SGT, UDP-glucose:sinapate glucosyltransferase; SCT, sinapoylglucose:choline sinapoyltransferase; SCE, sinapine esterase; SMT, sinapoylglucose:malate sinapoyltransferase. *Arabidopsis* mutant names are written in italics: *brt 1*, *bright trichomes 1*; *sng 1, 2*, *sinapoylglucose accumulator 1, 2*; *fah 1*, *ferulate hydroxylase 1*

It has been reported that three major sinapic acid esters, sinapoylglucose, sinapoylmalate, and sinapoylcholine, accumulate in *Arabidopsis* and other members of the *Brassicaceae* family (Fig. 1) [5, 6]. Sinapoylglucose, which is the immediate precursor of sinapoylcholine and sinapoylmalate that accumulate in seeds and leaves, is produced by a UDP-glucose: sinapic acid glucosyltransferase (SGT) that transfers the glucose moiety of UDP-glucose to the carboxyl group of sinapic acid [5]. The resulting 1-*O*-sinapoylglucose is a β-acetal ester that acts as an energy-rich acyl donor [7]; this donor provides the necessary free energy for the transacylation reaction catalyzed by sinapoylglucose:malate sinapoyltransferase (SMT) that generates sinapoylmalate in vegetative tissues [8]. In developing seeds, sinapoylglucose is also converted to sinapoylcholine by sinapoylglucose:choline sinapoyltransferase (SCT) [9–11]. Sinapoylcholine plays an important role during seed germination [12]. When the seeds start to germinate, the sinapoylcholine is hydrolyzed by sinapoylcholine esterase (SCE) to liberate sinapic acid and choline [12], and subsequently, the choline is oxidized to glycine betaine that functions as a stress-protecting agent by stabilizing proteins and membranes [13]. Thus, sinapoylcholine may serve as an important seed storage form of choline for the subsequent synthesis of phosphatidylcholine in developing seedlings. Currently, several *Arabidopsis* mutants have been identified to dissect the effects of **s**inapic acid ester accumulation at various stages of development on plant growth and yield (Fig. 1). For example, the *bright trichomes 1* (*brt1*) mutant, defective in *SGT*, showed a reduced epidermal fluorescence phenotype [9, 14]. The *Arabidopsis* mutant *sinapoylglucose accumulator 1* (*sng1*), defective in the gene encoding SMT, accumulated sinapoylglucose instead of sinapoylmalate in its leaves. Interestingly, the *Arabidopsis* loss-of-function mutant for *SCT* (*sng2*) was found to have increased sinapoylglucose content in the seeds and a corresponding decrease in the level of sinapoylcholine [5, 15, 16]. Additionally, the *FERULIC ACID HYDROXYLASE1* (*fah1*) and *REDUCED EPIDERMAL FLUORESCENCE1* (*ref1*) mutations lack sinapic acid and sinapate ester synthesis, respectively [9, 17–19].

It is well known that various stresses trigger the activation of the phenylpropanoid pathway in plants [20–22]. The phytohormone abscisic acid (ABA) is a major regulator of plant development and stress responses, including seed dormancy, germination, and drought resistance responses [23–25]. It is generally believed that both phenolic compounds and ABA act as inhibitors of plant growth and development, and they are involved in plant growth regulation [26]. In addition to their individual inhibitory actions, phenolic compounds have been demonstrated to antagonize some effects of ABA, for instance, reversing ABA-induced abscission, hypocotyl growth, and seed germination [27–29]. Earlier studies revealed that phenolic compounds such as *t*-cinnamic acid and *p*-coumaric acid reverse ABA-induced stomatal closure [30]. Furthermore, ABA also causes an increase in stomatal diffusive resistance that is recovered by *t*-cinnamic acid and *p*-coumaric acid [31]. Moreover, some phenolic compounds such as scopoletin and umbelliferone were found to be associated with substantial retention of K^+^ in guard cells, antagonizing the effect of ABA and ABA-mediated increases in epidermal diffusive resistance [32]. Conversely, hydrophenolic compounds were completely inactive [32]. Recently, the use of microarrays and quantitative proteomics has found that treatment with ABA alters the transcript levels of phenylpropanoid pathway genes in *Arabidopsis* suspension cells [20]. ABA also can activate the expression of *MYB10*, a transcription factor that plays a major role in the regulation of flavonoid/phenylpropanoid metabolism during ripening in *Fragaria x ananassa* fruit [33]. Together, these studies suggest that phenylpropanoid metabolism plays an important role in the response to ABA. It is possible, therefore, that phenolics affect plant growth and development by inhibiting ABA synthesis and signaling processes. However, direct biochemical and genetic evidence for this is lacking.

In this study, we investigated the roles of sinapic acid during seed germination in *Arabidopsis*. Our results show that sinapic acid is involved in regulating ABA-mediated inhibition of seed germination. To test the contribution of sinapic acid esters to the interaction with ABA, **s**inapic acid ester-accumulating *Arabidopsis* mutants were analyzed. Our findings suggest a novel model for the involvement of sinapic acid esters in ABA homeostasis during seed germination.

## Results

### Effects of sinapic acid on seed germination and early seedling growth

**Fig. 2:**
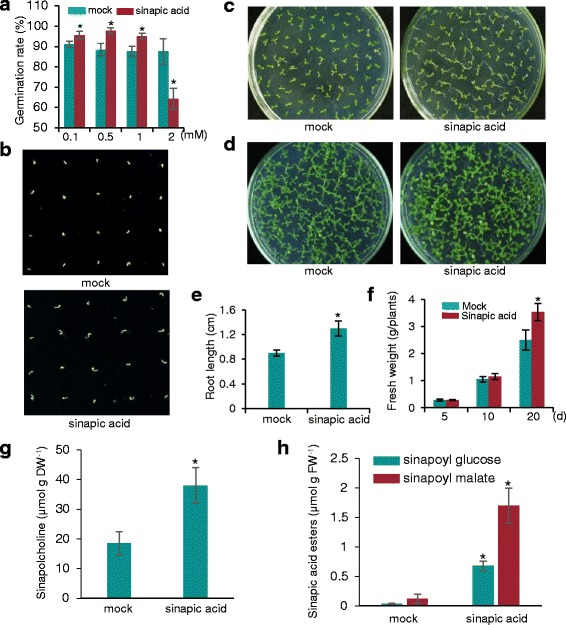
Sinapic acid promotes seed germination and early seedling growth in *Arabidopsis*. **a** Comparison of the germination rates of *Arabidopsis* seeds after exposure to different concentrations of sinapic acid for 2 d. Seeds were germinated and grown on MS medium with sinapic acid or mock-treatment (dimethyl sulfoxide, DMSO) as a control. **b**–**d** Seeds were germinated and grown on MS medium with 0.5 mM sinapic acid. The photographs were taken at 2 d (**b**), 8 d (**c**), and 20 d (**d**) after seed imbibition. **e** Quantitative analysis of root length after 0.5 mM sinapic acid treatment for 8 d. **f** Fresh weight (FW) biomass per five plants at different times for seedlings grown on MS medium with 0.5 mM sinapic acid. **g** Quantification of sinapoylcholine released from the wild-type seeds. After 2 d of treatment with 0.5 mM sinapic acid, sinapic acid esters were extracted from germinating seeds and sinapoylcholine was quantified by HPLC; DW, dry weight. **h** Sinapoylglucose and sinapoylmalate analyses in seedlings grown for 10 d after exogenous 0.5 mM sinapic acid treatment. Values are means ± SD of three independent experiments. Asterisks indicate significant changes compared with the mock (*P* < 0.05) calculated using Student’s *t*-test

As previously reported, sinapic acid esters are involved in protection against UV radiation, seed germination, and seedling development in brassicaceous plants [34]; it is, however, unclear how sinapic acid esters regulate seed germination. We, therefore, examined the role of sinapic acid in plant seed germination and early seedling development. First, we compared the germination rates of wild-type *Arabidopsis* seeds on Murashige and Skoog (MS) [35] medium containing different concentrations of sinapic acid. As shown in Fig. 2a, wild-type seed germination was promoted by sinapic acid concentrations ranging from 0.1 to 1 mM, with the germination rate of wild-type seeds in MS medium containing 0.5 mM sinapic acid increased by ~9.2% compared with the control (Fig. 2a, b). Next, we observed the effect of sinapic acid on root growth and early seedling development (Fig. 2c, d). Sinapic acid promoted root growth, causing an ~44% increase in root length compared with the mock (dimethyl sulfoxide was added, as the same volume of sinapic acid) treatment at 8 d after seed imbibition (Fig. 2c, e). Treatment with 0.5 mM sinapic acid for 20 d increased fresh seedling weight by ~20% compared with the mock treatment (Fig. 2f). To remove the effects of any trace of chemicals in the MS medium that could interfere with seed germination we also performed the germination assay on water medium. Freshly harvested *Arabidopsis* seeds were used in this study. The rate of seed germination increased by ~9% with 0.5 mM sinapic acid compared with the control, indicating that sinapic acid significantly promoted early seedling growth (Additional file 1: Figure S1). These findings are consistent with the seed germination results using MS medium. Together, these data suggest that sinapic acid is involved in seed germination and early plant development.

To test whether exogenous sinapic acid is converted into sinapic acid esters during seed germination in *Arabidopsis*, we analyzed sinapoylglucose and sinapoylcholine production using high-performance liquid chromatography (HPLC) mass spectrometry. *Arabidopsis* seeds were germinated on MS medium containing 0.5 mM sinapic acid. As shown in Fig. 2g, wild-type seeds, after imbibition with 0.5 mM sinapic acid for 2 d, accumulated ~36 μmol g^−1^ dry weight (DW) sinapoylcholine, while the control accumulated only ~17 μmol g^−1^ DW. When soluble phenolic compounds were extracted from seedlings after 20 d of growth, the levels of sinapoylglucose and sinapoylmalate following 0.5 mM sinapic acid treatment were two to three times higher than in the mock-treated seedlings (Fig. 2h). These results suggest that exogenous sinapic acid may be channeled into the phenylpropanoid pathway where it is subsequently converted into the corresponding sinapic acid esters by sinapoyltransferase. This could potentially support seed germination and increase the development of young seedlings.

### Effect of sinapic acid on ABA homeostasis

**Fig. 3:**
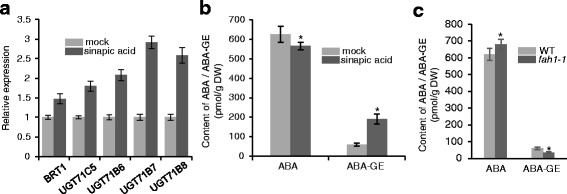
Analysis of the endogenous ABA and ABA- glucose ester (GE) levels following sinapic acid pretreatment of seeds in *Arabidopsis*. **a** qRT-PCR analyses of ABA UDP-glucosyltransferase genes after pretreatment of seeds with sinapic acid or DMSO (mock-treated control). The seeds were incubated with 0.5 mM sinapic acid or DMSO for 36 h at 4 °C in the dark and then germinated on MS agar medium for 1 d. Total RNAs were isolated from the treated and mock-treated seeds; *Actin2* primers were used in PCR as an internal control. **b** Analysis of endogenous ABA and ABA-GE levels using HPLC-mass spectrometry. Seeds were immersed in 0.5 mM sinapic acid or DMSO (mock) for 36 h at 4 °C in the dark and then transferred to MS medium for 1 d of germination. Data are based on three independent replicates (± SD). **c** Analysis of endogenous ABA and ABA-GE levels in *fah1–1 Arabidopsis* plants. Seeds were immersed in water for 36 h at 4 °C in the dark and then transferred to MS medium for 1 d of germination. Data are based on three independent replicates (± SD). Asterisks indicate significant changes compared with the control (*P* < 0.05) calculated using Student’s *t*-test

ABA is the key hormone associated with seed germination and vegetative growth and is known to interact with phenolic compound synthesis during plant development [20, 23–25]. To examine whether exogenous sinapic acid affects ABA homeostasis during seed germination, we first analyzed the expression of genes encoding UDP-glycosyltransferases (UGTs) and hydroxylases using real-time quantitative reverse transcriptase PCR (qRT-PCR). Previously reported UGTs UGT71C5, UGT71B6, UGT71B7, and UGT71B8, which displayed in vitro glucosylation activity toward ABA, belong to UGT subfamily 1 in *Arabidopsis* [36–38]. Under sinapic acid treatment, the transcript levels of *UGT71C5*, *UGT71B6*, *UGT71B7*, and *UGT71B8* were upregulated compared with the control (Fig. 3a). *UGT71B7* expression was upregulated more than 3-fold in seeds pretreated with sinapic acid for 12 h compared with the mock treatment. To further study whether sinapic acid affects ABA catabolism, we analyzed the expression of members of the *Arabidopsis* cytochrome P450 *CYP707A* gene family that encode proteins with ABA 8’-hydroxylase activity [39, 40]. There were no significant differences in the expression of *CYP707A1*, *2*, *3*, and *4* (Additional file 2: Figure S2a).

In addition to de novo ABA biosynthesis, it has been shown that the β-glucosidase (BG) homologs in *Arabidopsis,* β-glucosidase 1 (BG1) and BG2, generate ABA from ABA- glucose ester (GE) in the endoplasmic reticulum and vacuole, respectively [41, 42]. To determine whether sinapic acid affects hydroxylation of ABA-GE to ABA by the BGs, we analyzed the expression of *BG1* and *2* under sinapic acid treatment. As shown in Additional file 2: Figure S2B, there was no significant difference in the expression of *BG1* and *2* with sinapic acid, suggesting that exogenous sinapic acid mainly participates in the regulation of the endogenous level of free ABA by glucosylation, but does not affect ABA catabolism or hydroxylation of ABA-GE during seed germination.

To further demonstrate that sinapic acid affects ABA glucosylation, we examined the levels of free ABA and ABA-GE using reversed-phase HPLC in *Arabidopsis* seeds pretreated with sinapic acid for 48 h. We found that the level of ABA in the imbibed seeds was ~560 pmol g^−1^ after pretreatment with sinapic acid, compared with ~620 pmol g^−1^ after mock pretreatment. However, the level of ABA-GE in the seeds with sinapic acid pretreatment was ~190 pmol g^−1^, compared with ~60 pmol g^−1^ in the mock-treated seeds (Fig. 3b).

In *Arabidopsis*, *FAH1* encodes ferulate-5-hydroxylase, an enzyme in the phenylpropanoid pathway responsible for sinapic acid ester synthesis. The *fah1* mutant fails to accumulate sinapic acid esters [9]. To further confirm the degree of sinapic acid regulation of endogenous ABA and ABA-GE, free ABA and ABA-GE was measured in the null *fah1–1* mutant with decreased sinapic acid levels (Additional file 3: Figure S3). Consistently, the level of ABA reached ~680 pmol g^−1^ in *fah1–1* mutant seeds without sinapic acid treatment, compared with ~620 pmol g^−1^ in the wild-type seeds. Additionally, the level of ABA-GE was ~30 pmol g^−1^ in *fah1–1* compared with ~36 pmol g^−1^ in wild-type (Fig. 3c). Hence, our work provides genetic evidence that sinapic acid functions in ABA glucosylation during seed germination in *Arabidopsis*. Taken together, these findings suggest that sinapic acid influences ABA homeostasis by regulating ABA glucosylation in plants.

### Sinapic acid partly reversed ABA-mediated inhibition of *Arabidopsis* seed germination

**Fig. 4:**
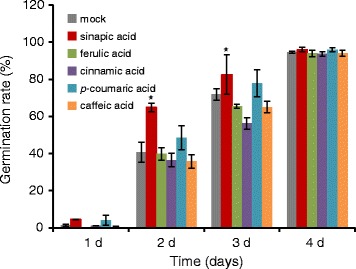
Sinapic acid decreased ABA-induced inhibition of seed germination in *Arabidopsis*. Wild-type *Arabidopsis* seeds were incubated with 0.5 mM sinapic acid, ferulic acid, cinnamic acid, *p*-coumaric acid, or caffeic acid for 36 h at 4 °C in the dark, and then germinated on MS agar medium with 0.2 μM ABA. Values are means ± SD of three independent experiments (>100 seeds per data point). Asterisks indicate significant changes compared with the control (*P* < 0.05) calculated using Student’s *t*-test

To further investigate the function of sinapic acid in the regulation of ABA homeostasis, we sowed *Arabidopsis* seeds onto a plate and soaked them in mock (dimethyl sulfoxide was added, as the same volume of sinapic acid) or 0.5 mM sinapic acid solutions at 4 °C in the dark. After 36 h incubation, the seeds were germinated in MS medium with 0.2 μM ABA. As shown in Fig. 4, a greater percentage of the seeds pretreated with sinapic acid germinated in MS medium containing ABA compared with the control; and 2 d after sowing, the germination percentage of seeds soaked in 0.5 mM sinapic acid was ~65%, while only ~40.5% of the mock-pretreated seeds had germinated. At 4 d, no significant differences were observed in germination percentage between sinapic acid-incubated seeds and mock-treated seeds (Fig. 4). These findings are consistent with sinapic acid affecting the balance between ABA and ABA-GE during seed germination, and further confirm that exogenous sinapic acid might be the primary phenolic compound that is channeled into seeds and converted into sinapic acid esters to regulate ABA homeostasis.

To evaluate whether the accumulation of phenylpropanoids has the same function as sinapic acid on seed germination in response to ABA, we examined the effects of cinnamic acid and its hydroxylated derivatives (caffeic acid, ferulic acid, p-coumaric acid, and *t*-cinnamic acid) on seed germination. As shown in Fig. 4, the pretreatment of seeds with caffeic acid, ferulic acid, or cinnamic acid did not result in antagonistic effects upon ABA-mediated inhibition of seed germination; conversely, these compounds slightly enhanced ABA inhibition of seed germination compared with the mock treatment. However, p-coumaric acid slightly increased seed germination upon ABA treatment.

### Loss of sinapic acid esters enhances susceptibility to ABA

**Fig. 5:**
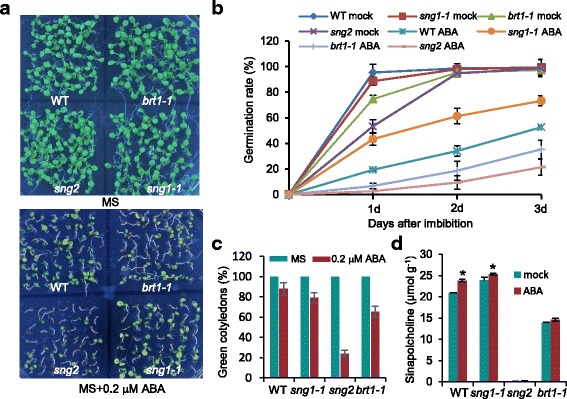
Effect of ABA on seed germination and early seedling growth in **s**inapic acid ester-accumulating mutants of *Arabidopsis*. **a** Eight-day-old wild-type, *sng1-1*, *sng2*, and *brt1-1* seedlings were grown in MS medium with or without 0.2 μM ABA. **b** Seed germination rates of wild-type (WT), *sng1-1*, *sng2*, and *brt1-1* in the presence of 0.2 μM ABA. **c** The number of green cotyledons was recorded for plants grown as in (**a**). **d** Quantification of sinapoylcholine released from 0.2 μM ABA pretreated seeds of wild-type and sinapic acid ester-accumulating mutants of *Arabidopsis*. Values are means ± SD of three independent experiments (>120 seeds per data point)

To provide further genetic evidence that sinapic acid interacts with ABA during seed germination and early plant growth, we analyzed mutants with impaired function of different enzymes involved in sinapic acid ester biosynthesis. First, the seed germination rate was determined in *sng2*, the null mutant for *SCT* [16]. Compared with the wild-type, seed germination of the *sng2* mutant was more sensitive to ABA, and its germination rate was reduced by 50% at 2 d (Fig. 5a, b). Additionally, the number of green cotyledons for *sng2* was reduced 4-fold compared with the wild-type at 8 d (Fig. 5a, c). *brt1-1* mutants, which are defective in SGT production, are responsible for the production of the sinapoyl donor sinapoylglucose [14]. Consistent with the role of *sng2*, *brt1-1* also reduced the rate of seed germination and the number of green cotyledons (Fig. 5). We also tested the seed germination rate in *sng1-1* mutants that are defective in *SMT* [15]. Interestingly, the seed germination rate of *sng1-1* was insensitive to ABA compared with the wild-type (Fig. 5b); moreover, there were no significant differences in the number of green cotyledons in *sng1-1* when compared with wild-type after 8 d of seedling growth (Fig. 5c).

To further investigate the relationship between sinapic acid ester biosynthesis and the ABA response during seed germination, we examined the sinapoylcholine level in *sng1-1*, *sng2*, and *brt1-1* mutants under ABA treatment. As shown in Fig. 5d, the level of sinapoylcholine in *sng1-1* and wild-type seeds was significantly higher following exposure to ABA than with the mock treatment. Almost no sinapoylcholine was found in *sng2*, however, and there were no significant differences between *brt1-1* and the wild-type. These findings suggest that the loss of *SCT* and *SGT* function resulted in a decrease in the levels of sinapoylcholine that might enhance susceptibility to ABA during seed germination.

### Sinapoylcholine might be a key sinapic acid ester in antagonizing the effect of ABA during seed germination

**Fig. 6:**
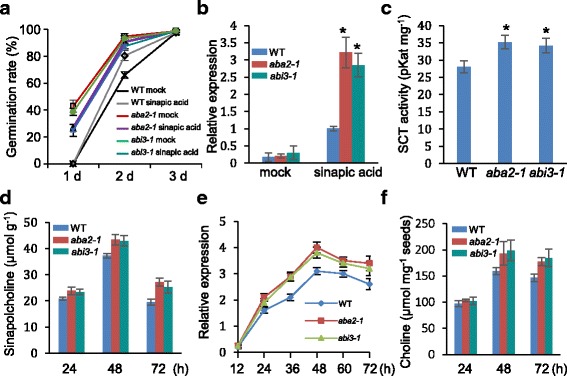
Effect of the *ABA2* and *ABI3* mutations on sinapic acid-mediated seed germination in *Arabidopsis*. **a** Germination of *aba2-1* and *abi3-1* seeds in the presence of 0.5 mM sinapic acid; WT, wild-type. **b** qRT-PCR analysis of *SCT* expression in the *aba2-1* and *abi3-1* mutant plants in response to sinapic acid. The seeds were incubated with 0.5 mM sinapic acid for 36 h at 4 °C in the dark and then germinated on MS medium for 1 d. Total RNAs were isolated from treated and untreated seedlings; *Actin2* primers were used in PCR as an internal control. **c** Analysis of SCT activity in *aba2-1* and *abi3-1* crude seed extracts. SCT activity was determined in the presence of sinapoylglucose and choline chloride as substrates. **d** Quantification of sinapoylcholine released from *aba2-1* and *abi3-1* seeds pretreated with 0.5 mM sinapic acid. **e** Expression of *SCE* in *aba2-1* and *abi3-1* mutant plants in response to 0.5 mM sinapic acid. **f** Quantification assay of free choline in wild-type, *aba2-1,* and *abi3-1* plants treated with 0.5 mM sinapic acid. Values are means ± SD of three independent experiments. Asterisks indicate significant changes compared with the control (*P* < 0.05) calculated using Student’s *t*-test

Mutants defective in ABA biosynthesis or catabolism have been shown to have reduced or increased seed dormancy. *ABSCISIC ACID DEFICIENT2* (*ABA2*) encodes a short-chain dehydrogenase/reductase family member in *Arabidopsis* that plays a unique and specific role in ABA biosynthesis [43]. The *ABA2*-deficient mutant *aba2-1* [44] was used to determine the link between sinapic acid and the ABA pathway during seed germination. The germination rate of *aba2-1* on MS medium lacking sinapic acid was ~27% at 1 d after imbibition. However, when the seeds were placed on MS medium containing 0.5 mM sinapic acid, the germination rate of *aba2-1* increased by ~15% compared with the mock treatment. For wild-type seeds, almost no germination had occurred at 1 d (Fig. 6a). To determine whether sinapic acid interferes with seed-specific ABA signal transduction, the role of *ABSCISIC ACID INSENSITIVE3* (*ABI3*) [45], a major regulator of seed maturation in *Arabidopsis,* was analyzed. As shown in Fig. 6a, the seed germination rate of the *Arabidopsis* loss of function mutant of *abi3-1* was also increased with 0.5 mM sinapic acid, compared with the mock treatment. These data are consistent with the greater insensitivity to sinapic acid shown by the *aba2-1* mutant (Fig. 6a).

To test whether sinapoylcholine is involved in ABA-mediated inhibition of seed germination, we performed qRT-PCR to examine *SCT* expression. With sinapic acid treatment, *SCT* expression was higher in the *aba2-1* and *abi3-1* mutants than in the wild-type (Fig. 6b). Moreover, SCT enzymatic activity, measured using extracts of sinapic acid-treated wild-type, *aba2-1,* and *abi3-1* seeds, was 27.02 ± 1.35, 34.22 ± 1.65, and 35.2 ± 2.1 pKat mg^−1^ protein, respectively (Fig. 6c). These data are consistent with the increased expression of *SCT* in *aba2-1* and *abi3-1* mutants.

Having found that sinapic acid led to the production of sinapoylcholine in pretreated seeds (Fig. 1g), we determined the sinapoylcholine content of the *aba2-1 and abi3-1* mutants. When the seeds of wild-type, *aba2-1,* and *abi3-1* were pretreated in 0.5 mM sinapic acid for 36 h before being germinated on MS medium for 48 h, the sinapoylcholine content of *aba2-1* and *abi3-1* had increased by ~2.58 and ~3.4 μmol g^−1^, respectively, compared with the wild-type (Fig. 6d). Therefore, we conclude that sinapoylcholine is responsible for ABA-mediated inhibition of seed germination.

To further investigate the physiological relevance of the interaction between sinapoylcholine and ABA, the expression of *SCE* was assayed in *aba2-1* and *abi3-1* mutants. As shown in Fig. 6e, *SCE* expression increased ~29 and ~23% in *aba2-1* and *abi3-1*, respectively, compared with wild-type. Similarly, free choline accumulation in germinating seeds also increased in the *aba2-1* and *abi3-1* mutants upon sinapic acid treatment (Fig. 6f). Next, we tested whether choline chloride increased seed germination. Similar to sinapic acid, choline chloride also increased seed germination (Additional file 4: Figure S4). These data suggest that sinapoylcholine metabolism might regulate ABA-mediated inhibition of *Arabidopsis* seed germination.

## Discussion

In this study, we investigated the role of sinapic acid in *Arabidopsis* seed germination and seedling growth using the *sng2* and *brt1-1* mutants. Our results showed that the sinapic acid ester metabolic pathways are involved in regulating ABA-mediated inhibition of seed germination and early seedling growth in *Arabidopsis*.

As mentioned above, phenylpropanoids suppress seed germination, cause root growth disorders, and inhibit plant growth [46]. Several known phenolic compounds such as cinnamic acid, flavonoids, and coumarins have been classified as natural inhibitors of plant growth regulation [26]. Sinapoylcholine is a member or derivative of the phenylpropanoid family that specifically accumulates in the seeds of cruciferous plants [34]. During seed germination, sinapoylcholine is hydrolyzed by SCE activity [47–50]. However, the physiological role of sinapoylcholine as a seed-specific ester is still unknown. In this study, we have shown that exogenous sinapic acid at concentrations of between ~0.1 and 1 mM could increase seed germination and the development of young seedlings (Fig. 2). A higher concentration of sinapic acid suppressed seed germination (Fig. 2a). When exogenous sinapic acid (0.5 mM) was applied to the medium, wild-type imbibed seeds and seedlings contained two to three times more of the sinapic acid esters sinapoylcholine and sinapoylglucose than did mock-treated control seedlings (Fig. 2g, h). Hence, we conclude that exogenous sinapic acid might be channeled into seeds where it is converted into sinapic acid esters, leading to the accumulation of these compounds in imbibed *Arabidopsis* seeds. This differs from the mechanisms of other phenolic compounds involved in the regulation of seed germination, root growth, and early seedling growth.

A number of phenolic compounds are known to antagonize the effects of ABA by, for instance, reversing ABA-induced abscission, hypocotyl growth, and seed germination [27, 29]. Some phenolic compounds, such as vanillic acid, gallic acid, salicylic acid, cinnamic acid, *p*-coumaric acid, ferulic acid, coumarin, chlorogenic acid, rutin, and morin antagonize ABA-induced stomatal closure [51]. Interestingly, all the benzoic acids, including sinapic acid, resulted in the recovery of ABA-induced stomatal closure [28]. These results suggest that phenolic compounds might be involved in ABA metabolism or ABA signaling in response to stress. This hypothesis was confirmed in assays examining the germination of wild-type seeds after pretreatment with sinapic acid that found that sinapic acid partly reversed the ABA-mediated inhibition of *Arabidopsis* seed germination (Fig. 4). However, pretreatment with several simple phenolic compounds such as *p*-coumaric acid, caffeic acid, and ferulic acid did not recover seed germination following ABA exposure (Fig. 4). Our data, therefore, reveal an important role for sinapic acid in regulating seed germination together with ABA, though other phenolic compounds do not mirror this relationship. These results strongly support the presence of a correlation between the accumulation of sinapic acid esters and ABA homeostasis during seed germination. Therefore, sinapic acid not only enables the recovery of ABA-induced stomatal closure [28] but may also antagonize ABA-mediated inhibition of seed germination.

As the metabolism of sinapic acid esters in seeds is controlled by the sinapoylglucose-dependent sinapoyltransferase UGT enzyme family that includes BRT1 (UGT84A2), it is possible that sinapic acid ester metabolism might be involved in ABA glucosylation. Indeed, expression of *BRT1* was induced by sinapic acid (Fig. 3a). Interestingly, the transcript levels of *UGT71B5*, *UGT71B6*, *UGT71B7*, and *UGT71B8* were apparently upregulated by sinapic acid compared with mock-treated samples (Fig. 3a). Conversely, the expression of *CYP707A* genes and *BG* genes were less affected by sinapic acid (Additional file 2: Figure S2). The endogenous ABA and ABA-GE levels determined using LC-mass spectrometry in this study confirm the hypothesis that sinapic acid plays a major role in ABA glucosylation. Exogenous sinapic acid treatment led to dynamic changes in endogenous ABA/ABA-GE concentrations (Fig. 3b), suggesting that sinapic acid may influence ABA homeostasis in germinating seeds. Accordingly, a loss of function mutation in *FAH1* also led to increased ABA-GE levels and reduced ABA levels (Fig. 3C). The genetic analysis showed that seed germination decreased the sensitivity to sinapic acid in *aba2* and *abi3* mutants compared with wild-type seeds (Fig. 6a) and that sinapic acid increased *SCT* gene expression and enzyme activity (Fig. 6b, c). Importantly, sinapic acid enhanced *SCE* gene expression and sinapoylcholine level. When exogenous sinapic acid was added, the level free choline increased along with seed germination (Fig. 6f). Hence, it is possible that exogenous sinapic acid is channeled via sinapoylglucose (1-*O*-sinapoyl-glucose) to sinapoylcholine, simultaneously enhancing free choline levels in the germinating seeds of *aba2-1* and *abi3-1* mutants, and antagonizing some effects of ABA-mediated inhibition of seed germination.

The involvement of sinapic acid or its derivatives in ABA-mediated inhibition of seed germination is also confirmed by *Arabidopsis* lines with mutations in the *SGT*, *SCT*, and *SMT* genes. As illustrated in Fig. 1, the *brt1-1* mutation impairs the gross metabolic flux toward sinapoylmalate in leaves or sinapoylcholine in seeds [52]. The *sng2* mutant accumulated a high level of sinapoylglucose in its seeds, corresponding to a decreased level of sinapoylcholine [5, 15, 16]. The fact that *sng2* and *brt1-1* seed germination exhibited greater sensitivity to ABA than seed germination in wild-type suggests that sinapoylcholine plays an important role in ABA-regulated seed germination (Fig. 5). Furthermore, when the seeds were planted on MS medium containing 0.2 μM ABA, the *sng1-1* mutant was found to be less sensitive to ABA than the wild-type, *sng2,* or *brt1-1* (Fig. 5). One possibility is that the *Arabidopsis sng1-1* mutant accumulated sinapoylglucose instead of sinapoylmalate in the imbibed seeds, corresponding to an increased level of sinapoylcholine and resulting in a blocking of ABA synthesis and breaking of seed dormancy. These results were also partly confirmed by an assay of the sinapoylcholine content of the *sng1-1* mutant (Fig. 5d). Consistently, defects in the ABA pathway increased sinapic acid-induced seed germination and *SCT* expression (Fig. 6a, c).

Overall, the accumulation of sinapoylcholine in the seed is a typical characteristic of many members of the *Brassicaceae* family. Once seeds begin to germinate, sinapoylcholine is mobilized by SCE hydrolysis to liberate sinapic acid and choline for germinating seedlings. Therefore, it is possible that the accumulation of sinapic acid and choline disturbs ABA homeostasis during seed germination. However, the link between sinapic acid-induced free choline accumulation and ABA-mediated inhibition of seed germination is further strengthened by our observations.

## Conclusion

We demonstrate that sinapic acid esters might regulate ABA-mediated inhibition of dormancy breakage, germination, and growth in *Arabidopsis*. Hence, our investigation highlights the importance of sinapic acid metabolism in the conjugation cycle of ABA in ABA homeostasis during seed germination. Further research is needed to ascertain how sinapic acid esters regulate seed germination through a negative feedback loop modulating ABA homeostasis.

## Methods

### Plant material and growth conditions

*Arabidopsis thaliana* ecotype Columbia was used in this study. The *sng1-1* [15], *sng2* [16], and *brt1-1* [14] homozygous mutants (Col-0) were generously provided by Clint Chapple (Purdue University, USA). *fah1–2* (CS6172) and *abi3-1* (CS24) were obtained from the *Arabidopsis* Biological Resource Center (Ohio State University). *aba2-1*/*eas1–1* was screened by chlorophyll fluorescence imaging in our lab [44]. For seed germination, all seeds were sterilized and kept for 2 d at 4 °C in the dark to break dormancy. The seeds were then placed on 0.6% agar-containing MS medium (PhytoTech) with different levels of phenolic compounds or ABA as indicated, or the seeds were placed on wet filter paper (water medium) with sinapic acid or water alone as a control. The plates were incubated at 22 ± 2 °C with a 16-h-light photoperiod.

### Pretreatment of *Arabidopsis* seeds

Freshly harvested *Arabidopsis* seeds were surface-sterilized with 0.1% mercuric chloride for 3 min, washed three times with sterile water before sowing, and then incubated in 0.5 mM sinapic acid (50 mM sinapic acid stock solution in 100% dimethyl sulfoxide) or the corresponding control for 36 h in the dark at room temperature. For the germination assay, the pre-treated seeds were then sown on solid MS medium with or without 0.2 μM ABA at 4 °C for 2 d in the dark before being transferred into a growth chamber with a 16/8 h (24/18 °C) day/night cycle with a light intensity of 150 μmol m^−2^ s^−1^. The number of germinated seeds was recorded daily over 5 d. The experiment was carried out with three replicates, each with a group of 100 seeds per treatment.

### RNA extraction and qRT-PCR

Total RNA was extracted using the RNeasy plant mini kit (Qiagen) according to the manufacturer’s instructions. RNA samples were quantified with a Nanodrop spectrophotometer (ND-1000; Labtech). Reverse transcription was performed with 3 μg of total RNA and M-MLV (Promega) according to the manufacturer’s instructions. All gene expression experiments were repeated at least three times (2 × SYBR Green Realtime Master Mix, Novoprotein). PCR primer sequences are presented in Additional file 5: Table S1.

### Determination of ABA and ABA-GE content

Samples of imbibed seeds before solid-phase extraction were treated as described previously [37]. Assays of ABA and ABA-GE were performed essentially as described by Liu [38]. Briefly, 1 ml of pretreated sample with 20 ml of internal standard solution (chloromycetin, 14.426 ng ml^−1^) was loaded into a Kinetex 2.6 μm C18 Column (50 × 2.1 mm). Samples were eluted with 2 ml of buffer containing methanol: distilled deionized water: acetic acid (80:19:1, *v*/v/v). The eluent was dried at 40 °C under a gentle stream of nitrogen. The residue was reconstituted by the addition of 200 ml of methanol: water: acetic acid (45:54:1, *v*/v/v); 10 ml aliquots of supernatant were analyzed by LC-mass spectrometry (AB Sciex API QTRAP4000).

### Analysis of sinapic acid esters

Rosette leaves and pretreated seeds of *Arabidopsis* were ground into powder in liquid nitrogen and extracted overnight at 4 °C with 80% methanol containing 25 μM chrysin as an internal standard. Samples were ground briefly and then centrifuged at 13,000×*g* for 10 min. The imbibed seed extracts were prepared from 1 mg of seeds suspended in 0.1 ml of 80% methanol. Sinapic acid ester contents were determined by HPLC (Agilent). The sample was resolved on a Kinetex 2.6 μm C18 Column (50 × 2.1 mm) in 0.2% acetic acid (A) with an increasing concentration gradient of acetonitrile containing 0.2% acetic acid (B) at a constant rate of 0.8 ml min^−1^: 0–20 min, 30% B; 20–25 min, 100% B. UV absorption was monitored at 330 nm using a multiple-wavelength photodiode array detector (Agilent). Peaks were identified and quantified using commercially available standard substances.

### Determination of SCT activity

Enzyme extraction and assay conditions were based on those used previously to purify and assay SCT from *Arabidopsis* [53]. To assay the crude seed extracts, wild-type, *aba2-1*, and *abi3-1* seeds were frozen in liquid nitrogen and each ground to a fine powder. This powder was stirred for 20 min in five volumes of 100 mM potassium phosphate buffer (pH 6.8) containing 20 mM NaCl and 4% *w*/*v* insoluble polyvinylpolypyrrolidone. The samples were filtered through Miracloth (Calbiochem, La Jolla, CA, USA) and centrifuged for 20 min at 14,000×*g*. The supernatant was added to 0.1% *w*/*v* protamine sulfate, stirred for 20 min, and centrifuged for 20 min at 14,000×*g*. The supernatant was again filtered through Miracloth (Calbiochem), and the protein was precipitated by adding ammonium sulfate to 85% saturation, followed by centrifugation at 14,000×*g* for 20 min. The pellet was resuspended in 100 mM potassium phosphate buffer (pH 7.0) containing 50 mM NaCl, desalted into 100 mM potassium phosphate buffer (pH 7.0) using PD-10 Sephadex G-25 M columns (Supelco, Bellefonte, PA, USA), and used for the determination of SCT activity. Each assay contained 50 ml of 2.5 mM sinapoylglucose, 10 ml of 100 mM choline chloride, and 30 ml of protein extract. The assays were incubated for 60 min at 30 °C, stopped by the addition of 400 ml of cold 50% methanol, and analyzed by HPLC (Agilent).

### Choline assay

Free choline from germinating seeds was assayed using a Choline Assay Kit (Abnova) in accordance with the manufacturer’s instructions. For an assay of crude seed extracts, wild-type, *aba2-1,* and *abi3-1* seeds were frozen in liquid nitrogen and each ground to a fine powder. This powder was stirred in cold PBS buffer (pH 6.8) for 1 h. The samples were filtered through Miracloth (Calbiochem) and centrifuged for 5 min at 14,000×*g*. For each sample, 300 μl of supernatant was transferred to a clean tube and neutralized with 50 μl 6 M NaOH. The neutralized supernatant was then assayed by spectrophotometer (Thermo Fisher).

## Additional files

Additional file 1: Figure S1. Sinapic acid promotes seed germination and early seedling growth in *Arabidopsis*. **a** Comparison of germination rates of *Arabidopsis* after exposure to different concentrations of sinapic acid for 2 d. Fresh seeds were germinated and grown on wet filter paper with 0.5 mM sinapic acid or water alone as a control. Values are means ± SD of three independent experiments. Asterisks indicate significant changes compared with the mock (*P* < 0.05) calculated using Student’s *t*-test. **b** Photographs taken 7 d after seed imbibition. Seeds were germinated and grown on water alone, or water with 0.5 mM sinapic acid. (PPTM 396 kb)

Additional file 2: Figure S2. Expression of ABA metabolism genes in response to sinapic acid. **a** Quantitative real-time RT-PCR (qRT-PCR) to examine the expression of ABA catabolism genes (*CYP707A1*, *CYP707A2*, *CYP707A3*, and *CYP707A4*) with sinapic acid. **b** qRT-PCR to examine the expression of de novo ABA biosynthesis genes (*BG1* and *BG2*) with sinapic acid. The seeds were incubated with 0.5 mM sinapic acid or dimethyl sulfoxide (DMSO) for 36 h at 4 °C in the dark and then germinated on MS agar medium for 1 d. Total RNA was isolated from the treated and mock-treated seeds; *Actin2* primers were used as an internal control. (PPTM 107 kb)

Additional file 3: Figure S3. Quantification of sinapoylcholine released from wild-type and *fah1–1* seeds pretreated with 0.5 mM sinapic acid. After 2 d of treatment with 0.5 mM sinapic acid, sinapic acid esters were extracted from seeds and sinapoylcholine was quantified by HPLC; DW, dry weight. (PPTM 58 kb)

Additional file 4: Figure S4. Germination of *aba2-1* and *abi3-1* seeds in the presence of 400 mg l^−1^ choline chloride. (PPTM 69 kb)

Additional file 5: Table S1. List of primers used in this study. (DOCX 14 kb)
