# Supplementary material for: Sinapic acid or its derivatives interfere with abscisic acid homeostasis during Arabidopsis thaliana seed germination
Source: BMC Plant Biol. 2017 Jun 6;17:99. doi: 10.1186/s12870-017-1048-9 (PMC5461752; doi:10.1186/s12870-017-1048-9)
Supplement: Supplementary file 1 — Sinapic acid promotes seed germination and early seedling growth in Arabidopsis. a Comparison of germination rates of Arabidopsis after exposure to different concentrations of sinapic acid for 2 d. Fresh seeds were germinated and grown on wet filter paper with 0.5 mM sinapic acid or water alone as a control. Values are means ± SD of three independent experiments. Asterisks indicate significant changes compared with the mock (P < 0.05) calculated using Student’s t-test. b Photographs taken 7 d after seed imbibition. Seeds were germinated and grown on water alone, or water with 0.5 mM sinapic acid. (PPTM 396 kb) [file 12870_2017_1048_MOESM1_ESM.pptm]

## Slide 1
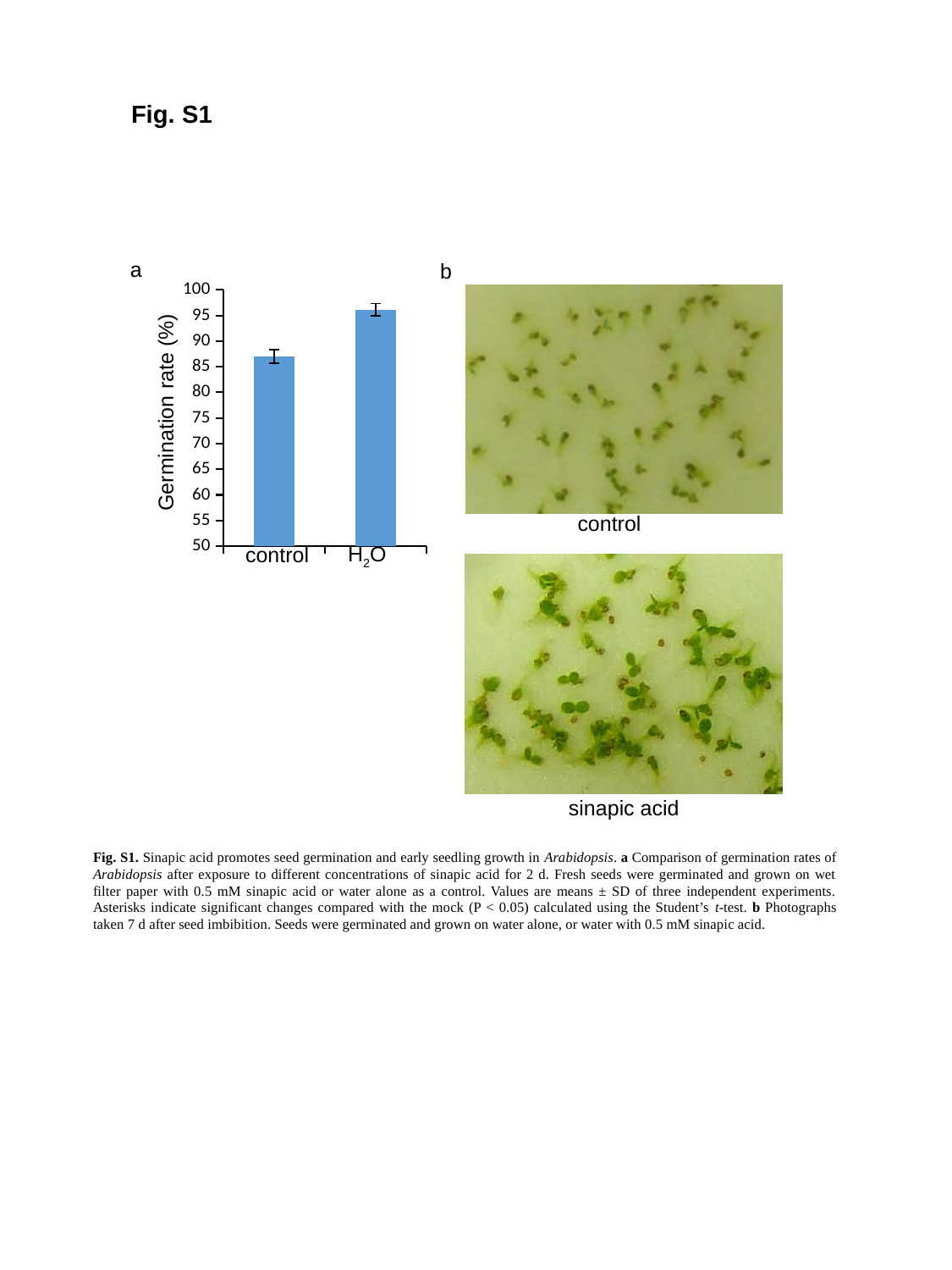

Fig. S1
a
b
### Chart
| Category | |
|---|---|Germination rate (%)
control
H2O
control
sinapic acid
Fig. S1. Sinapic acid promotes seed germination and early seedling growth in Arabidopsis. a Comparison of germination rates of Arabidopsis after exposure to different concentrations of sinapic acid for 2 d. Fresh seeds were germinated and grown on wet filter paper with 0.5 mM sinapic acid or water alone as a control. Values are means ± SD of three independent experiments. Asterisks indicate significant changes compared with the mock (P < 0.05) calculated using the Student’s t-test. b Photographs taken 7 d after seed imbibition. Seeds were germinated and grown on water alone, or water with 0.5 mM sinapic acid.
