# Supplementary material for: Sinapic acid or its derivatives interfere with abscisic acid homeostasis during Arabidopsis thaliana seed germination
Source: BMC Plant Biol. 2017 Jun 6;17:99. doi: 10.1186/s12870-017-1048-9 (PMC5461752; doi:10.1186/s12870-017-1048-9)
Supplement: Supplementary file 2 — Expression of ABA metabolism genes in response to sinapic acid. a Quantitative real-time RT-PCR (qRT-PCR) to examine the expression of ABA catabolism genes (CYP707A1, CYP707A2, CYP707A3, and CYP707A4) with sinapic acid. b qRT-PCR to examine the expression of de novo ABA biosynthesis genes (BG1 and BG2) with sinapic acid. The seeds were incubated with 0.5 mM sinapic acid or dimethyl sulfoxide (DMSO) for 36 h at 4 °C in the dark and then germinated on MS agar medium for 1 d. Total RNA was isolated from the treated and mock-treated seeds; Actin2 primers were used as an internal control. (PPTM 107 kb) [file 12870_2017_1048_MOESM2_ESM.pptm]

## Slide 1
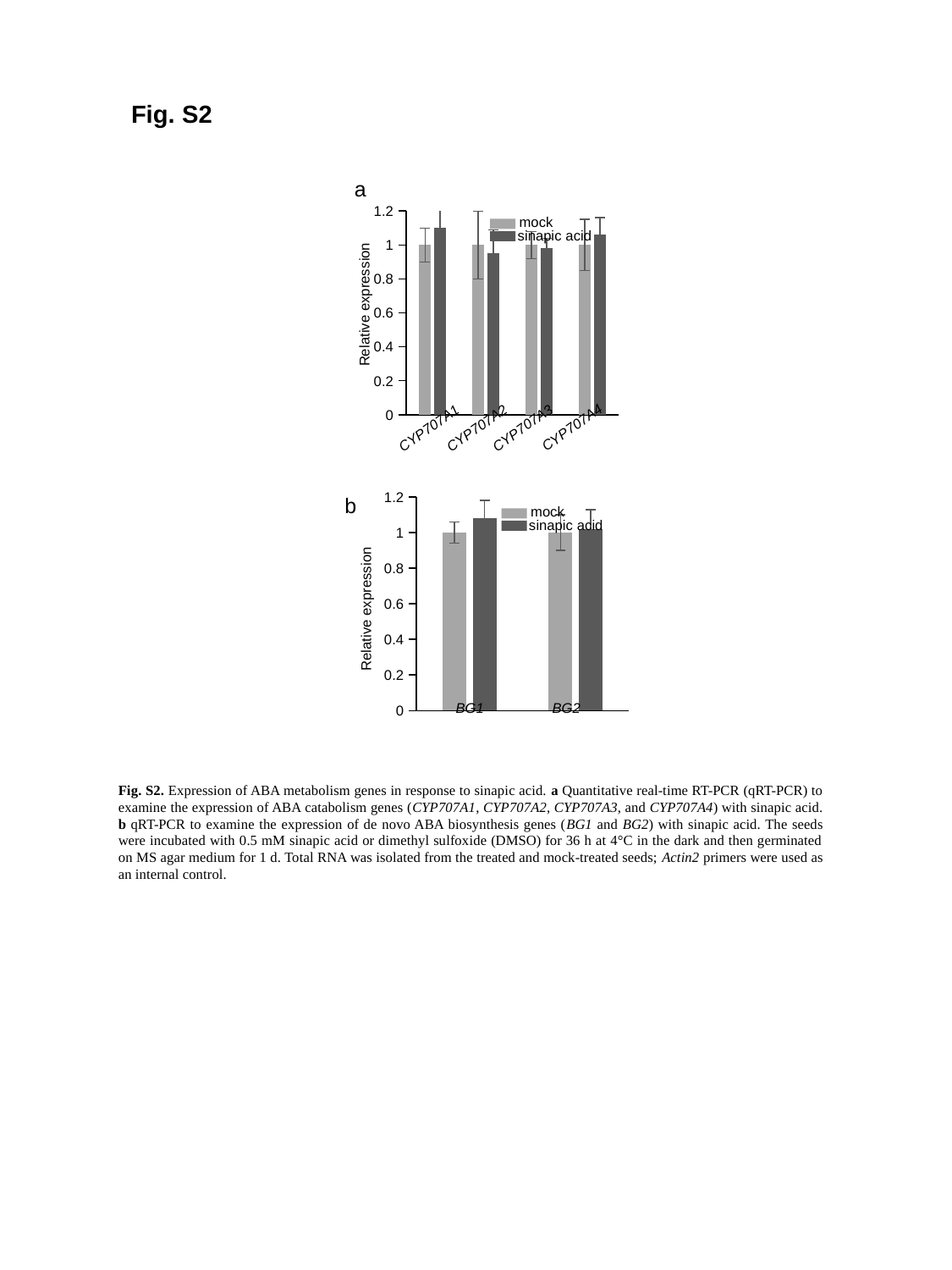

Fig. S2
a
### Chart
| Category | | |
|---|---|---|mock
sinapic acid
Relative expression
CYP707A4
CYP707A3
CYP707A1
CYP707A2
### Chart
| Category | | |
|---|---|---|b
mock
sinapic acid
Relative expression
BG1
BG2
Fig. S2. Expression of ABA metabolism genes in response to sinapic acid. a Quantitative real-time RT-PCR (qRT-PCR) to examine the expression of ABA catabolism genes (CYP707A1, CYP707A2, CYP707A3, and CYP707A4) with sinapic acid. b qRT-PCR to examine the expression of de novo ABA biosynthesis genes (BG1 and BG2) with sinapic acid. The seeds were incubated with 0.5 mM sinapic acid or dimethyl sulfoxide (DMSO) for 36 h at 4°C in the dark and then germinated on MS agar medium for 1 d. Total RNA was isolated from the treated and mock-treated seeds; Actin2 primers were used as an internal control.
