# Supplementary material for: Sinapic acid or its derivatives interfere with abscisic acid homeostasis during Arabidopsis thaliana seed germination
Source: BMC Plant Biol. 2017 Jun 6;17:99. doi: 10.1186/s12870-017-1048-9 (PMC5461752; doi:10.1186/s12870-017-1048-9)
Supplement: Supplementary file 3 — Quantification of sinapoylcholine released from wild-type and fah1–1 seeds pretreated with 0.5 mM sinapic acid. After 2 d of treatment with 0.5 mM sinapic acid, sinapic acid esters were extracted from seeds and sinapoylcholine was quantified by HPLC; DW, dry weight. (PPTM 58 kb) [file 12870_2017_1048_MOESM3_ESM.pptm]

## Slide 1
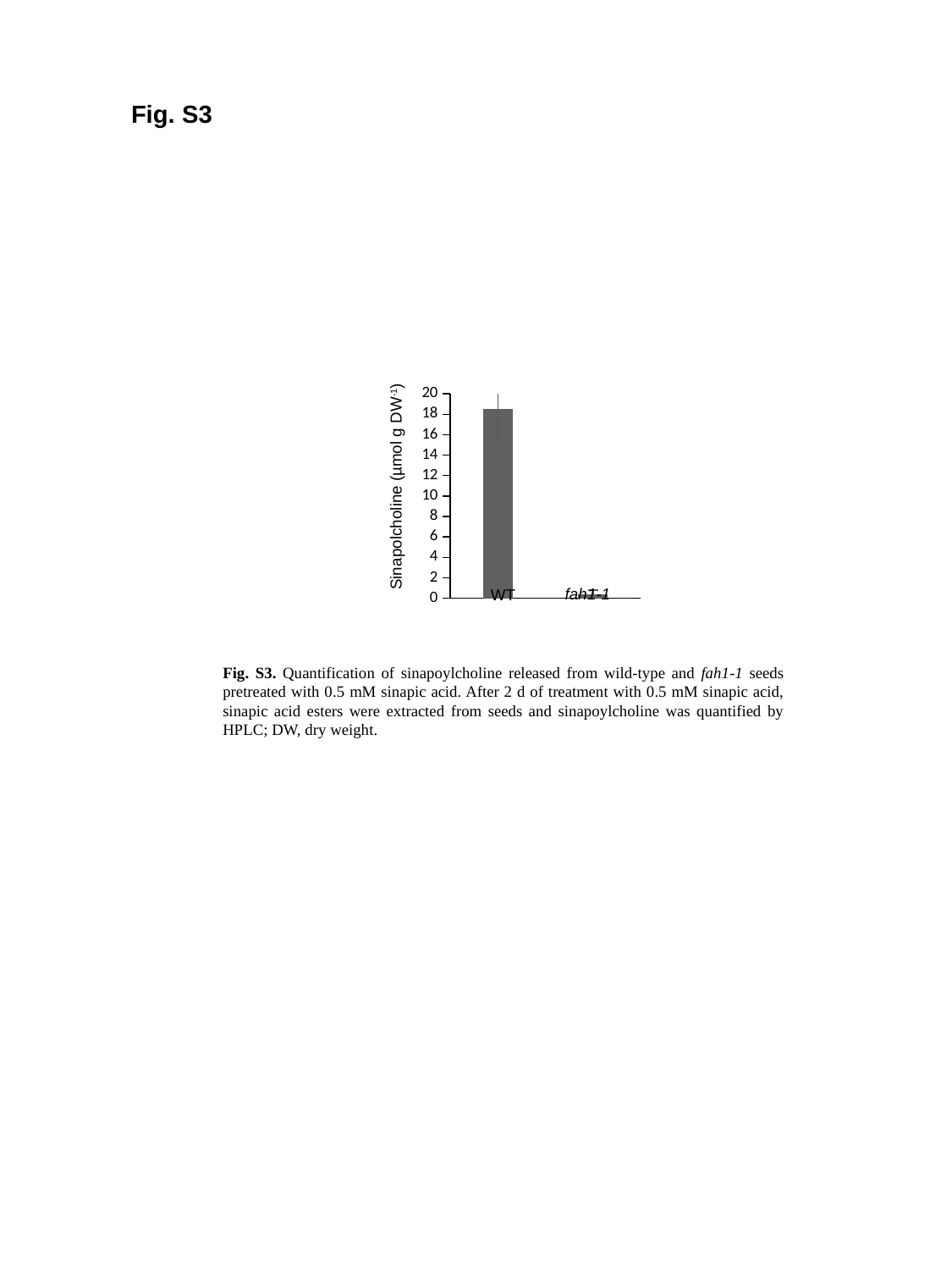

Fig. S3
### Chart
| Category | |
|---|---|Sinapolcholine (µmol g DW-1)
fah1-1
WT
Fig. S3. Quantification of sinapoylcholine released from wild-type and fah1-1 seeds pretreated with 0.5 mM sinapic acid. After 2 d of treatment with 0.5 mM sinapic acid, sinapic acid esters were extracted from seeds and sinapoylcholine was quantified by HPLC; DW, dry weight.
