# Supplementary material for: Sinapic acid or its derivatives interfere with abscisic acid homeostasis during Arabidopsis thaliana seed germination
Source: BMC Plant Biol. 2017 Jun 6;17:99. doi: 10.1186/s12870-017-1048-9 (PMC5461752; doi:10.1186/s12870-017-1048-9)
Supplement: Supplementary file 4 — Germination of aba2-1 and abi3-1 seeds in the presence of 400 mg l−1 choline chloride. (PPTM 69 kb) [file 12870_2017_1048_MOESM4_ESM.pptm]

## Slide 1
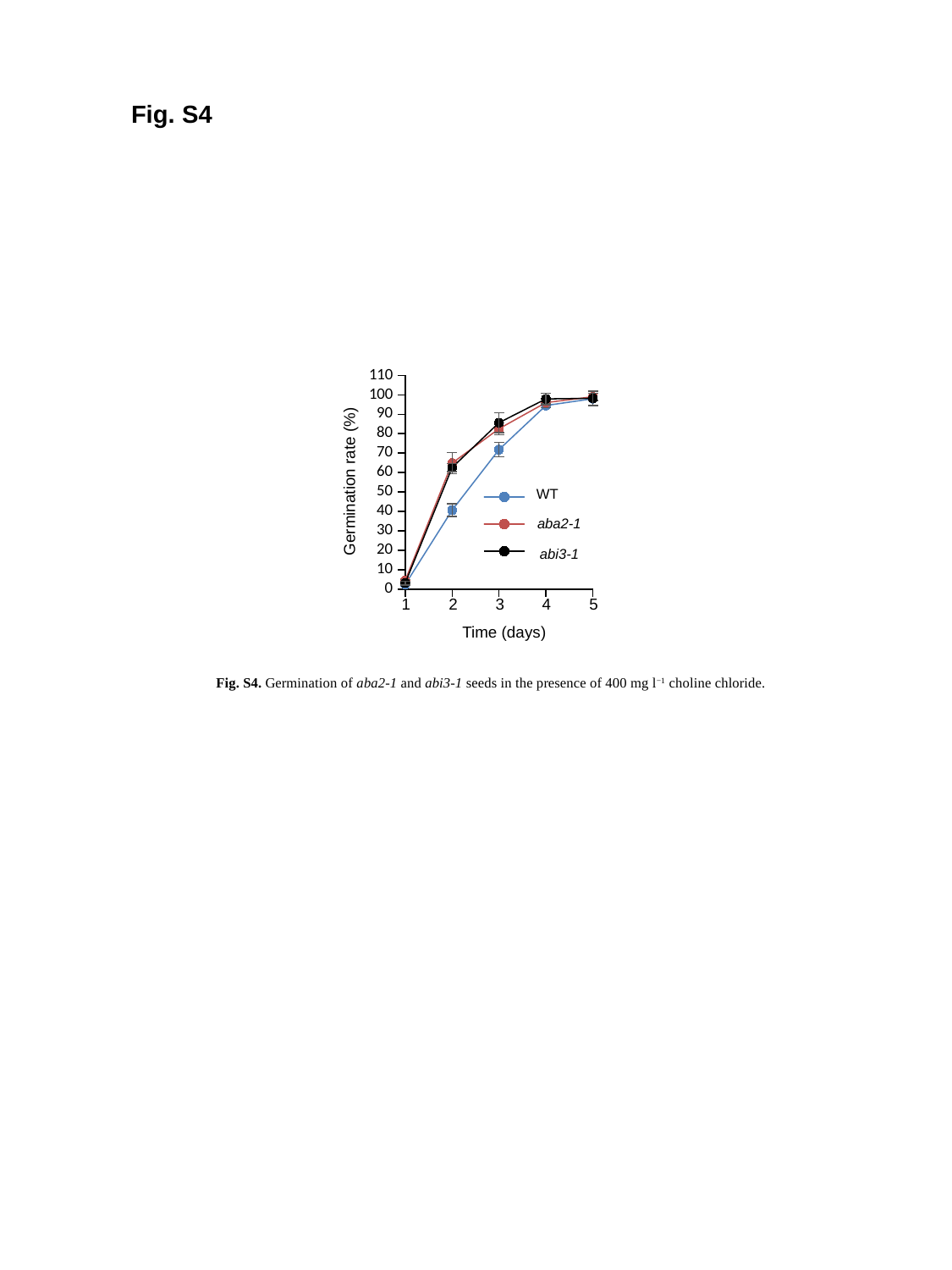

Fig. S4
### Chart
| Category | | | |
|---|---|---|---|Germination rate (%)
WT
aba2-1
abi3-1
1
2
3
4
5
Time (days)
Fig. S4. Germination of aba2-1 and abi3-1 seeds in the presence of 400 mg l−1 choline chloride.
