# Supplementary material for: Sinapic acid or its derivatives interfere with abscisic acid homeostasis during Arabidopsis thaliana seed germination
Source: BMC Plant Biol. 2017 Jun 6;17:99. doi: 10.1186/s12870-017-1048-9 (PMC5461752; doi:10.1186/s12870-017-1048-9)
Supplement: Supplementary file 5 — List of primers used in this study. (DOCX 14 kb) [file 12870_2017_1048_MOESM5_ESM.docx]

**Table S1**. List of primers used in this study.

| GENEs | Primers |
| --- | --- |
| *BRT11* | Forward: 5'- GGTAAAGGCTATCTCCGGTATG -3' |
|  | Reverse: 5'- GTGGTCGGAGGATGGTTAAG-3' |
| *UGT71C5* | Forward: 5'- ATCCGGGTCTAGCTTCGG-3' |
|  | Reverse: 5'- ATTCCACGGCCCATTGTT-3' |
| *UGT71B6* | Forward: 5'- TCGAGATGGTGGAAGAGC-3'  Reverse: 5'- GTTTCCGACCAAGCAATA-3' |
| *UGT71B7* | Forward: 5'- AAGTCGGTGCTTCCGATTA C -3'  Reverse: 5'- GTCATCTCGATGGTT GGTTGA-3' |
| *UGT71B8* | Forward: 5'- CGGTGATAGACGTGGCTAATG-3' |
|  | Reverse: 5'- CACTGACACTGTACTCCTTCTTATC-3' |
| *CYP707A1* | Forward: 5'- CTCACTCTCTTCGCCGGAAG-3'  Reverse: 5'- GGAGGGAGTGGGAGTTTGGAA-3' |
| *CYP707A2* | Forward: 5'- CGTCTCTCACATCGAGCTCCTT-3'  Reverse: 5'- CCAAAAGTCCATCAACACCCTC-3' |
| *CYP707A3* | Forward: 5'- CTCTGTTTCTCTGTTTACTCCGATTTA-3'  Reverse: 5'- CGTATCTTCTGTTTTGCTGCA-3' |
| *CYP707A4* | Forward: 5'- CCTGAAACCATCCGTAAACTCAT-3'  Reverse: 5'- TTGGCCCAAGATTGTAAGGAA-3' |
| *AtBG1* | Forward: 5'- TTACTATACTTCAGTGTTTGCAAAAG-3'  Reverse: 5'- CTAGAGTTCTTCCCTCAGCTTG -3' |
| *AtBG2* | Forward: 5'- GTGCTAAAAAGGGTTCTG-3'  Reverse: 5'- CAGTGAGATACCAACAC-3' |
| *SCT* | Forward: 5'- GCTGGCTCTGGATACTCTTATG-3'  Reverse: 5'- GGGTGTTTCACAAACCAACTC-3' |
| *SCE* | Forward: 5'- TTAACCACCTTCCTCAATCCGCCT-3'  Reverse: 5'- CAGTTGCACCATACACCGCGAAAT-3' |
| *ACTIN1* | Forward: 5'- GGTAACATTGTGCTCAGTGGTGG -3'  Reverse: 5'- AACGACCTTAATCTTCATGCTGC -3' |
